# New insights into metabolic dysfunction-associated steatotic liver disease and oxidative balance score

**DOI:** 10.3389/fnut.2023.1320238

**Published:** 2024-01-05

**Authors:** Lei Peng, Lurong Li, Jiahao Liu, Yuanyuan Li

**Affiliations:** ^1^Department of Gastroenterology, First Affiliated Hospital of Nanjing Medical University, Nanjing, China; ^2^Department of Endocrinology, Children's Hospital of Nanjing Medical University, Nanjing, China

**Keywords:** metabolic dysfunction-associated steatotic liver disease (MASLD), oxidative balance score (OBS), nutrition, NHANES, mortality

## Abstract

**Background:**

The relationship between oxidative stress and metabolic dysfunction-associated steatotic liver disease (MASLD) has not been studied, which remains inadequately recognized. This is a cross-sectional study in a US adult population to explore the relationship between MASLD and oxidative balance scores (OBS), which containing integrating dietary nutrition and lifestyle factors.

**Methods:**

We analyzed data from National Health and Nutrition Examination Survey during 2017–2018. Multivariate logistic regression, restricted cubic spline curve (RCS) and subgroup analysis were used to investigate the association between OBS and MASLD. Cox regression analysis was utilized to assess the association between OBS and all-cause mortality among individuals.

**Results:**

The multivariable-adjusted odds ratio (OR) and 95% confidence interval (CI) for the highest quartile of OBS (Q4) was 0.30 (0.12, 0.77) (*p* = 0.012) compared to the lowest quartile of OBS (Q1). The RCS regression and subgroup analysis indicated an inverted relationship between OBS and the development of MASLD. The OBS Q4 group (HR: 0.15, 95% CI: 0.03–0.87; *p* = 0.035) exhibited a lower risk of all-cause death than the Q1 group.

**Conclusion:**

OBS is statistically significantly and negatively correlated with the risk of MASLD and all-cause mortality in US adults. More prospective investigations are required to substantiate our findings.

## Introduction

Non-alcoholic fatty liver disease (NAFLD) is a clinically common chronic liver disease that pathologically manifests as excessive lipid deposition in liver parenchymal cells (1). With changing lifestyles, NAFLD is becoming prevalent worldwide, and approximately 25% of adults are affected by this disease (2, 3). NAFLD can progress to the development of severe hepatic disease or even liver cell carcinoma, with a lack of effective treatment and a poor prognosis at late stages (4). NAFLD has an indisputable onset and is often asymptomatic in its early stages and is often not appreciated; therefore, early prevention, diagnosis and intervention of NAFLD are needed to slow its progression (5). Following the Delphi Consensus process, the term “metabolic dysfunction-associated steatohepatitis” (MASLD) became the successor to the term “NAFLD” (6) to enhance the precision of nomenclature.

MASLD is a highly heterogeneous disease in which multiple factors, such as genetics, environment, aging, and poor dietary practice, especially the over-intake of high-calorie diets abundant in saturated fatty acids or simple carbohydrates, may be involved in its pathophysiological mechanisms (7). Thus, changing dietary behavior and diet structure is a feasible strategy for the prevention and treatment of MASLD (8). It has been shown that changes in oxidative stress indicators occur in patients with NAFLD, including elevated oxidative stress marker 8-oxo-dG (9) and decreased coenzyme Q10 (10), suggesting that oxidative stress may exert an essential part in the pathophysiological process of MASLD. Therefore, antioxidants in food, including certain antioxidant vitamins and micronutrients, exhibit some antioxidant stress activity and may be effective in preventing and treating MASLD (11, 12). Although studies have shown a relationship between oxidative stress-related nutrients/foods and MASLD, it is possible that dietary interfactions may interfere with the effectiveness of integrated nutrients (13). Therefore, a new strategy based on dietary models related to oxidative and antioxidant modification is needed.

The oxidative balance score (OBS) evaluates an individual’s level of oxidative stress by integrating dietary nutrition and lifestyle (14). The OBS was first proposed in 2002 in an epidemiologic study of a Belgian male smoking population, which used vitamin C and β-carotene as the antioxidant constituents and iron as the pro-oxidant constituent and showed that the lower the OBS score was, the higher the level of oxidative homeostasis in an individual, indicating that the lower the oxidative homeostasis score was, the higher the mortality rate in the population (15). Thereafter, numerous studies were conducted to investigate the relationship between OBS and a wide range of chronic disorders, such as type 2 diabetes mellitus (16), cardiovascular disease (17), and colorectal cancer (18). Recently, a study conducted in Iran showed that the greater the adherence to an OBS, the lower the odds of developing NAFLD (19).

Therefore, cross-sectional research based on the 2017–2018 National Health and Nutrition Examination Survey (NHANES) was carried out to evaluate the association between OBS and the degree of hepatosteatosis as measured by quantification of vibration-controlled transient elastography (VCTE) in US adults.

## Materials and methods

### Study design and data source

The current study is a retrospective, cross-sectional, population-based analysis of data extracted from the 2017–2018 US National Health and Nutrition Examination Survey (NHANES) database, which is intended to provide an assessment of the dietary state of health and nutrition using a sophisticated, multiphasic design to collect and analyze data that are representative of the nation as a whole. An extensive assessment process was required for participants, including a household interview and a mobile examination center (MEC) visit, which consisted of a medical physical examination, specialized measurements, and laboratory tests (20). In this study, liver ultrasound transient elastography (TE) examinations were first used, and participants supplied two 24-h periods of detailed dietary intake information, subsequently used to estimate energy, nutrient and other food component intake. Initial dietary memories were collected over the course of the NHANES visit, and the subsequent ones were gathered over the phone 3–10 days afterwards.

### Study population

**Figure 1:**
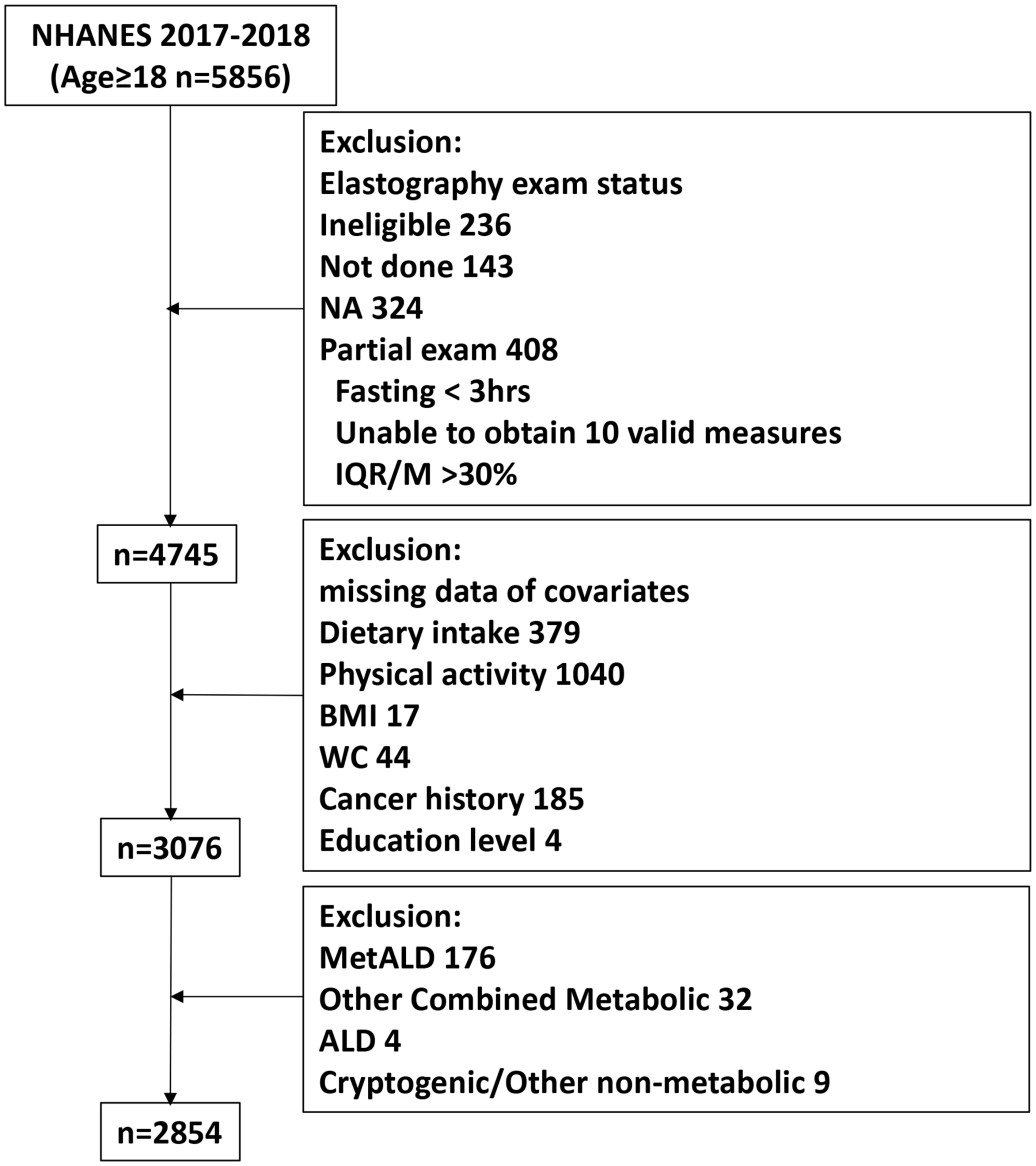
Flowchart of the study participants.

The present study was originally comprised of 5,856 participants aged ≥18 years. Of these, 4,745 underwent complete elastography. In addition, participants with the following conditions were excluded: (1) missing data (dietary intake information, exercise data, body mass index, waist circumference, cancer history, and education level);(2) MASLD with increased alcohol intake (MetALD), defined as a daily intake of 20–50 g of alcohol for females and 30–60 g daily for males; (3) alcoholic liver disease (ALD), defined as a daily intake of more than 50 g of alcohol for females and more than 60 g daily for males; (4) other underlying etiologies of hepatic disease. Finally, we recruited 2,854 participants in this study (Figure 1).

### Study variables

#### Measurement of MASLD, MASH and liver fibrosis

The severity of liver steatosis was evaluated by the controlled attenuation parameter (CAP) derived from FibroScan®, which is available at the Mobile Examination Center (MEC) using vibration-controlled transient elastography (VCTE) technology (21). According to a previous study, the definition of SLD is a median CAP ≥263 dB/m (22). The SLD participants meet one of the 5 cardiometabolic risk factors as suggested on the recent Delphi consensus statement (23) and excluded MetALD, ALD, underlying etiologies of hepatic disease were defined as MASLD. The definition we used to classify metabolic dysfunction-associated steatohepatitis (MASH) is ALT >25 U/L or AST > 25 U/L on the basis of MASLD (24). Liver stiffness measurements (LSM) determined clinically significant and advanced fibrosis with cut-offs of 8.6 kPa and 13.1 kPa based on Youden’s study, respectively (25).

#### Oxidative balance score

In the present study, we obtain the OBS for each individual participant. According to Azita’s study (19), we selected 10 dietary components (selenium, fiber, β-carotene, folic acid, iron, vitamin D, vitamin C, vitamin E, saturated fatty acids, and polyunsaturated fatty acids) and lifestyle components (physical activity, smoking and obesity) for their association with oxidative stress (Table 1). Besides, the coffee/tea consumption and non-steroidal anti-inflammatory drug (NSAID) using participant as important modulator of oxidant balance (26, 27), we added them to the lifestyle components. The dietary components were categorized into quintiles. In the case of dietary antioxidants, the first through fifth quintiles were scored as 0–4. A reversed scoring method was used for dietary pro-oxidants. For NSAID using, we assigned 0: never, 4: regular user. For physical activity, we assigned 0: MET-minute/week≤400, 2: 400 < MET-minute/week ≤1000, and 4: MET-minute/week>1,000. For BMI and WC, we gave the following scores: 0 for BMI ≥ 30 kg/m^2^ and male WC ≥ 102 cm/female WC ≥ 88 cm; 2 for BMI ≥ 30 kg/m^2^ or male WC ≥ 102 cm/female WC ≥ 88 cm; and 4 for BMI < 30 kg/m^2^ and male WC < 102/female WC < 88 cm. With regard to smoking, the scale was as follows: 0 for current smoker (smoked >100 cigarettes in a lifetime and smoked on several days or every day), 2 for ex-smoker (smoked >100 cigarettes in a lifetime and currently do not smoke), and 4 for never smoker (smoked <100 cigarettes in a lifetime). The scores were then added together to calculate the OBS for each subject.

**Table 1:** Oxidative balance score assignment scheme.

OBS components	Property		Scoring assignment				
			0	1	2	3	4
Lifestyle components							
Physical activity (MET-minute/week)	Antioxidant		≤400		400–1,000		≥1,000
BMI and WC	Prooxidant	Male	BMI ≥ 30 + WC ≥ 102		BMI ≥ 30 or WC ≥ 102		BMI < 30 + WC < 102
		Female	BMI ≥ 30 + WC ≥ 88		BMI ≥ 30 or WC ≥ 88		BMI < 30 + WC < 88
Smoking	Prooxidant		current smoking		former smoking		never smoking
NSAID use	Antioxidant		Never				Regular
Caffeine (mg/d)	Antioxidant		Quintile1	Quintile2	Quintile3	Quintile4	Quintile5
Dietary components							
Dietary fiber (g/d)	Antioxidant		Quintile1	Quintile2	Quintile3	Quintile4	Quintile5
Carotene (RE/d)	Antioxidant		Quintile1	Quintile2	Quintile3	Quintile4	Quintile5
Total folate (mcg/d)	Antioxidant		Quintile1	Quintile2	Quintile3	Quintile4	Quintile5
Vitamin C (mg/d)	Antioxidant		Quintile1	Quintile2	Quintile3	Quintile4	Quintile5
Vitamin E (ATE) (mg/d)	Antioxidant		Quintile1	Quintile2	Quintile3	Quintile4	Quintile5
Selenium (mcg/d)	Antioxidant		Quintile1	Quintile2	Quintile3	Quintile4	Quintile5
Vitamin D (mcg/d)	Antioxidant		Quintile1	Quintile2	Quintile3	Quintile4	Quintile5
SFA (g/d)	Prooxidant		Quintile5	Quintile4	Quintile3	Quintile2	Quintile1
PUFA (g/d)	Prooxidant		Quintile5	Quintile4	Quintile3	Quintile2	Quintile1
Iron (mg/d)	Prooxidant		Quintile5	Quintile4	Quintile3	Quintile2	Quintile1

OBS, Oxidative Balance Score; MET, metabolic equivalent; BMI, body mass index; WC, waist circumference; SFA, saturated fatty acids; PUFA, polyunsaturated fatty acids.

#### Covariables

Drawing on existing publications and clinical considerations, a selection of covariates that may serve as potential confounders in the relationship between OBS and MASLD was made. Through standardized household interviews, we obtained demographic features, including age, gender, ethnicity, educational level, and financial condition. Educational level was classified into several categories: below high school, high school graduate/GED or equivalent, and college or above. Financial condition was categorized as low (PIR ≤ 1.0), medium (1 < PIR ≤ 4), and high (PIR > 4). To be evaluated for diabetes, subjects were required to fulfill one of the following criteria: (1) history of insulin use, (2) clinically previous diagnosis of diabetes, (3) taking glucose-lowering medication, and (4) laboratory data HbA1c ≥ 6.5% and fasting blood glucose ≥126 mg/dL (28). Inclusion criteria for hypertensive patients were as follows: (1) Subjects were required to answer at least one of the following questions by answering “(1) Be ever told on two or more occasions that they have hypertension or (2) Whether they have ever been told about their hypertension and taken prescribed medication” and obtaining at least one affirmative answer. (2) Subjects were identified as having hypertension by a clinical measurement of blood pressure with a mean of three consecutive systolic blood pressure measurements ≥140 mmHg or a mean of three consecutive diastolic blood pressure measurements ≥90 mmHg. Multiple imputation was performed by R for missing covariables measured at the baseline visit according to a previous study (29).

### Statistical analysis

We used weighted samples, strata, and subgroups from the NHANES database and calculated weighted populations for this research using the SURVEY program in SAS. Continuous variables were presented in the form of weighted means and standard errors, with categorical variables presented in the form of unweighted numbers and weighted proportions. We performed logistic regression analysis using SURVEYLOGISTIC statements to examine the relationship between OBS and MASLD. We performed multivariate regression analyses to adjust for potential confounders identified in single-variable regressions, consisting of age in years, sex, race, education level, and comorbidities (history of diabetes, hypertension, and cancer). Statistical analyses of categorical variables were performed using weighted *χ*2 tests, and analyses of continuous variables were performed using weighted linear regression models to identify between-group differences. Stratified multivariate regression analysis was used for subgroup analysis. It is also recommended to use the RCS function when adjusting for continuous exposure to minimize residual confounding (30). In this study, a RCS model with four nodes was used to investigate the relationship between OBS and MASLD prior to and after adjustment for confounders. All analyses were performed using the R program and EmpowerStats. *p* values less than 0.05 were considered statistically significant.

## Results

### Participant characteristics

A total of 2,854 participants from NHANES (2017–2018) were included in this study, of whom 1,294 (45.34%) were diagnosed with MASLD based on a previous study. Among all participants with MASLD, the prevalence was higher in males (56.67%) than in females (43.33%) (*p* = 0.019). Patients who had MASLD were much older and were more likely to be non-Hispanic white, with no differences in educational attainment or income between these two groups (Table 2). Baseline comparison of characteristics indicated that patients with MASLD had a greater prevalence of diabetes mellitus (21.72% vs. 4.38%, *p* < 0.001), hypertension (62.12% vs. 37.03%, *p* < 0.001), and smoking (41.79% vs. 39.63%, *p* = 0.009). The MASLD patients also had higher BMIs and waist circumferences. Participants without MASLD had significantly greater OBS (*p* < 0.001) in comparison to participants suffering from MASLD. Comparing laboratory characteristics, we found that ALT, GGT, HSCRP, and cholesterol were significantly higher in the MASLD group, while serum albumin was significantly lower (Table 2). When we separate the MASLD into MASH and MASLD^1^(MASLD without MASH)(27.44%) based on ALT and AST, the incidence of MASH was 17.90%. The OBS was decreased in MASH group (Supplementary Table S1). However, when compared participants with clinically significant fibrosis (12.75%) and advanced fibrosis (3.63%) to non-fibrosis, OBS showed no difference in the population (*p* = 0.237) (Supplementary Table S2).

**Table 2:** Baseline characteristics of all participants by MASLD.

Weighted characteristics of the study population				
Characteristic	Total	MASLD		Value of *p*
		No	Yes	
*N*	2,854	1,560	1,294	
Weighted *N*	15,36,53,090	8,81,27,629	6,55,25,460	
Age (years)	46 ± 1	43 ± 1	50 ± 1	<0.001
Gender (%)				0.019
Male	51.61	47.84	56.67	
Female	48.39	52.16	43.33	
Race (%)				<0.001
Mexican American	9.15	6.58	12.59	
Other Hispanic	6.82	7.06	6.51	
Non-Hispanic White	62.49	63.88	60.61	
Non-Hispanic Black	10.62	12.24	8.44	
Others	10.92	10.24	11.84	
Education level (%)				0.165
<high school	9.11	8.31	10.18	
High school graduate/GED or equivalent	26.92	25.54	28.76	
College graduate or above	63.98	66.14	61.06	
Economic status (%)				0.292
Low (PIR ≤ 1)	12.01	12.43	11.45	
Middle (1 < PIR < 4)	48.31	46.33	51.00	
High (PIR ≥ 4)	39.68	41.24	37.55	
Diabetes (%)				<0.001
Yes	11.77	4.38	21.72	
No	88.23	95.62	78.28	
Hypertension (%)				<0.001
Yes	47.73	37.03	62.12	
No	52.27	62.97	37.88	
History of cancer (%)				0.026
Yes	8.99	7.61	10.85	
No	91.01	92.39	89.15	
Smoking status (%)				0.009
Current	16.94	18.57	14.74	
Former	23.62	21.06	27.05	
Never	59.44	60.37	58.21	
NSAID use				<0.001
Never	83.35	88.87	75.93	
Regular	16.65	11.13	24.07	
BMI (kg/m2)	29.1 ± 0.3	26.2 ± 0.3	33.0 ± 0.4	<0.001
Waist circumference (cm)	99.1 ± 0.7	91.2 ± 0.7	109.6 ± 0.8	<0.001
Physical activity (Met.h/wk)	1,377 ± 54	1,423 ± 64	1,315 ± 59	0.091
OBS	30.10 ± 0.33	31.03 ± 0.44	28.85 ± 0.28	<0.001
OBS				<0.001
Q1	20.87	16.32	26.99	
Q2	26.54	24.89	28.77	
Q3	23.76	24.46	22.82	
Q4	28.83	34.33	21.43	
Laboratory features				
ALT (U/L)	23 ± 1	20 ± 1	27 ± 1	<0.001
AST (U/L)	22 ± 0	22 ± 1	23 ± 0	0.403
GGT(IU/L)	27 ± 1	23 ± 1	33 ± 1	<0.001
Total Bilirubin (mg/dL)	0.5 ± 0.0	0.5 ± 0.0	0.5 ± 0.0	0.150
Albumin (g/dL)	4.14 ± 0.01	4.16 ± 0.02	4.10 ± 0.02	0.006
HSCRP (mg/L)	3.58 ± 0.23	2.82 ± 0.35	4.58 ± 0.18	<0.001
Cholesterol(mg/dL)	190 ± 2	187 ± 1	194 ± 3	0.025

All values represented are weighted means ± SE or weighted percentage. The value of *p* was calculated by a weighted linear regression model and weighted chi-square test, respectively.

SE, standard error; PIR, poverty income ratio; BMI, body mass index; WC, waist circumference; MET, metabolic equivalent; OBS, oxidative balance score; ALT, alanine aminotransferase; AST, aspartate aminotransferase; GGT, gamma glutamyl transferase; HSCRP, hypersensitive c-reactive protein.

Then, all 2,854 individuals were divided into four groups based on OBS quartiles: group 1 (OBS Q1, OBS: 14 to 25), group 2 (OBS Q2, OBS: 26 to 29), group 3 (OBS Q3, OBS: 30 to 33), and group 4 (OBS Q4, OBS: 34 to 47). Individuals in the 1st quartile (Q1) with lower OBS (as a reference group) were more likely to be younger, women, white, less educated, and with less income. Furthermore, participants in Q1 were more likely to smoke and be less physically active. As OBS quartiles progressively increase, BMI progressively decreases. Compared with those in the lowest quartile of OBS, subjects in the highest quartile had lower levels of GGT, HSCRP and higher level of albumin (Table 3). Serum GGT (31) and albumin (32) are considered prooxidant and antioxidant indicators in previous studies, respectively. From OBS Q1 to Q4, GGT gradually decreased (*p* = 0.019) and albumin gradually increased (*p* < 0.001).

**Table 3:** Baseline characteristics of all participants by the OBS quartile.

Characteristic	Total	Q1	Q2	Q3	Q4	Value of *p*
		14–25	26–29	30–33	34–47	
*N*	Total	678	687	674	815	
Age (years)	46 ± 1	43 ± 1	45 ± 1	48 ± 1	48 ± 1	0.001
Gender (%)						0.003
Male	51.61	41.73	51.31	51.63	59.01	
Female	48.39	58.27	48.69	48.37	40.99	
Race (%)						<0.001
Mexican American	9.15	10.20	8.21	10.31	8.29	
Other Hispanic	6.82	4.73	6.19	6.25	9.40	
Non-Hispanic White	62.49	58.20	65.30	62.38	63.08	
Non-Hispanic Black	10.62	16.81	10.84	10.32	6.19	
Others	10.92	10.06	9.46	10.74	13.04	
Education level (%)						<0.001
<high school	9.11	13.58	10.23	7.33	6.30	
High school graduate/GED or equivalent	26.92	38.92	26.41	26.75	18.82	
College graduate or above	63.98	47.49	63.36	65.91	74.88	
Economic status (%)						<0.001
Low(PIR ≤ 1)	12.01	16.45	14.39	11.62	7.08	
Middle(1 < PIR < 4)	48.31	53.95	50.89	44.95	44.71	
High(PIR ≥ 4)	39.68	29.60	34.73	43.43	48.22	
Diabetes (%)						0.254
Yes	11.77	14.73	10.36	9.53	12.79	
No	88.23	85.27	89.64	90.47	87.21	
Hypertension (%)						0.245
Yes	47.73	48.69	50.84	49.01	43.12	
No	52.27	51.31	49.16	50.99	56.88	
History of cancer (%)						0.279
Yes	8.99	8.35	7.19	9.34	10.83	
No	91.01	91.65	92.81	90.66	89.17	
Smoking status (%)						<0.001
Current	16.94	32.93	21.26	11.79	5.63	
Former	23.62	27.86	22.32	24.45	21.05	
Never	59.44	39.22	56.42	63.76	73.32	
NSAID use						<0.001
Never	83.35	94.81	85.44	80.98	75.08	
Regular	16.65	5.19	14.56	19.02	24.92	
BMI(kg/m2)	29.1 ± 0.3	32.2 ± 0.6	30.0 ± 0.5	28.2 ± 0.4	26.8 ± 0.3	<0.001
Waist circumference (cm)	99.1 ± 0.7	106.0 ± 1.1	100.9 ± 1.2	97.4 ± 1.2	93.7 ± 0.8	<0.001
Physical activity (Met.h/wk)	1,377 ± 54	1,228 ± 63	1,325 ± 75	1,294 ± 83	1,601 ± 91	0.009
Laboratory features						
ALT (U/L)	23 ± 1	23 ± 1	26 ± 2	21 ± 1	23 ± 1	0.086
AST (U/L)	22 ± 0	21 ± 1	24 ± 1	21 ± 0	23 ± 1	0.112
GGT(IU/L)	27 ± 1	31 ± 2	29 ± 2	27 ± 2	24 ± 1	0.019
Total Bilirubin (mg/dL)	0.5 ± 0.0	0.4 ± 0.0	0.5 ± 0.0	0.5 ± 0.0	0.5 ± 0.0	0.002
Albumin (g/dL)	4.14 ± 0.01	4.05 ± 0.03	4.14 ± 0.02	4.15 ± 0.01	4.18 ± 0.02	<0.001
HSCRP (mg/L)	3.58 ± 0.23	4.95 ± 0.34	3.98 ± 0.29	3.72 ± 0.71	2.11 ± 0.13	<0.001
Cholesterol(mg/dL)	190 ± 2	190 ± 6	190 ± 2	194 ± 2	187 ± 3	0.341

All values represented are weighted means ± SE or weighted percentage. The value of *p* was calculated by a weighted linear regression model and weighted chi-square test, respectively.

SE, standard error; PIR, poverty income ratio; BMI, body mass index; WC, waist circumference; MET, metabolic equivalent; OBS, oxidative balance score; ALT, alanine aminotransferase; AST, aspartate aminotransferase; GGT, gamma glutamyl transferase; HSCRP, hypersensitive c-reactive protein.

### Relationship between OBS and MASLD/liver fibrosis

Multivariate logistic regression indicated that OBS was statistically correlated with MASLD (Table 4). In model 2, we found that each 1-unit increase in OBS resulted in a 7% reduction in the risk of MASLD (OR = 0.93, 95% confidence interval (CI) 0.90–0.95), suggesting that OBS was negatively associated with the risk of MASLD. In the crude model and model 1, the highest quartile was significantly associated with MASLD compared with the lowest quartile (*p* for trend<0.001, <0.001). In addition, adherence to the 4th quartile of OBS was also found to be correlated with MASLD after adjusting for confounders in model 2 (OR = 0.30, 95% CI 0.12–0.77; *p* = 0.012, *p* for trend = 0.001). However, OBS (continuous and categorical) was not associated with the risk of clinically significant fibrosis and advanced fibrosis in the all models (Supplementary Table S3).

**Table 4:** Association of the OBS with MASLD.

OBS	OR (95% CI); value of *p*					
	Crude model		Model 1		Model 2	
Continuous	0.94(0.92,0.96)	<0.001	0.92(0.89,0.94)	<0.001	0.93(0.90,0.95)	0.003
Q1	1.00 (ref)		1.00 (ref)		1.00 (ref)	
Q2	0.70(0.51,0.95)	0.043	0.63(0.47,0.85)	0.014	0.68(0.36,1.26)	0.073
Q3	0.56(0.38,0.83)	0.014	0.45(0.30,0.67)	0.004	0.53(0.22,1.27)	0.052
Q4	0.38(0.26,0.55)	<0.001	0.27(0.18,0.41)	<0.001	0.30(0.12,0.77)	0.012
*p* for trend		<0.001		<0.001		0.001

Model 1: adjusted for age, gender, and race.

Model 2: Model 1+ education level, diabetes, hypertension, cancer history and serum albumin.

The OBS was converted from a continuous variable to a categorical variable (quartiles). Weighted logistic regression was used to obtain OR, 95% CI and *p* for trend.

Crude model was adjusted with no covariates.

### Subgroup analysis

Group analyses were performed based on age, gender, race, education level, and history of diabetes, hypertension and cancer (Table 5). Regarding the race and education subgroup, statistical significance was found (interaction *p* = 0.008 and < 0.001). When stratified by race, among non-Hispanic whites, we found that OBS was associated with a lower prevalence of MASLD, whereas in other races, this trend was less pronounced than that in non-Hispanic whites. When considering education level, the negative association between OBS and MASLD was more pronounced among those with college or higher education. However, age stratification, gender subgroup, history of diabetes, HBP, and cancer had no significant effect on the results of the analysis (Table 5).

**Table 5:** Subgroup analyses of the association between OBS and MASLD.

Subgroup analyses of the association between OBS and NAFLD, NHANES 2017–2018						
Variables	Q1	Q2	Q3	Q4	*p* for trend	*p* for interaction
Age group						0.178
<60	Ref	0.80 (0.60, 1.07)	0.61 (0.40, 0.93)	0.37 (0.24, 0.56)	0.011	
≥60	Ref	0.39 (0.19, 0.78)	0.43 (0.17, 1.11)	0.24 (0.14, 0.42)	0.017	
Gender						0.316
Male	Ref	0.81 (0.54, 1.23)	0.82 (0.52, 1.31)	0.42 (0.26, 0.69)	0.034	
Female	Ref	0.64 (0.45, 0.92)	0.42 (0.23, 0.76)	0.29 (0.16, 0.52)	0.014	
Race						0.008
Non-Hispanic White	Ref	0.63 (0.39, 1.00)	0.59 (0.34, 1.01)	0.22 (0.14, 0.37)	0.007	
Others	Ref	0.81 (0.55, 1.20)	0.55 (0.39, 0.77)	0.63 (0.38, 1.04)	0.096	
Education level						<0.001
<high school	Ref	0.66 (0.26, 1.63)	0.66 (0.31, 1.40)	0.49 (0.19, 1.25)	0.259	
High school graduate/GED or equivalent	Ref	0.42 (0.23, 0.75)	0.89 (0.49, 1.63)	0.43 (0.20, 0.92)	0.215	
College graduate or above	Ref	0.85 (0.54, 1.33)	0.46 (0.28, 0.77)	0.30 (0.19, 0.48)	0.012	
Diabetes						0.944
Yes	Ref	0.82 (0.32, 2.06)	0.67 (0.24, 1.86)	0.42 (0.19, 0.92)	0.079	
No	Ref	0.69 (0.53, 0.91)	0.57 (0.37, 0.90)	0.34 (0.22, 0.50)	0.008	
Hypertension						0.879
Yes	Ref	0.78 (0.56, 1.09)	0.61 (0.40, 0.93)	0.35 (0.23, 0.53)	0.013	
No	Ref	0.63 (0.41, 0.98)	0.56 (0.28, 1.12)	0.34 (0.21, 0.56)	0.019	
History of cancer						0.141
Yes	Ref	0.69 (0.10, 4.68)	0.19 (0.04, 0.85)	0.16 (0.04, 0.67)	0.034	
No	Ref	0.70 (0.56, 0.89)	0.64 (0.45, 0.93)	0.37 (0.24, 0.57)	0.015	

Data are presented as OR (95% CI). Adjusted for age, gender, race, education level, diabetes, hypertension, cancer history, and albumin. In different stratified policies, stratified variables were not adjusted themselves.

### RCS analysis

**Figure 2:**
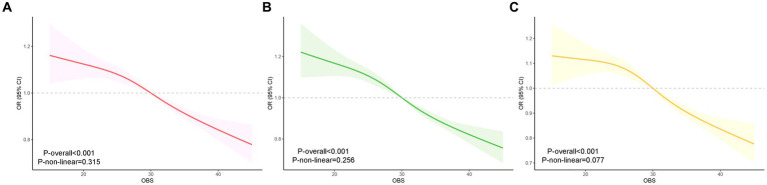
RCS analysis of the association between OBS and MASLD. Crude model was adjusted with no covariates. Model 1: adjusted for age, gender and race. Model 2: in addition to the variables in Model 1, education level, diabetes, hypertension, cancer history, and albumin were added. RCS curve of the association between OBS and MASLD of crude model **(A)**, Model 1 **(B)** and Model 2 **(C)**. RCS, restricted cubic spline.

We further assessed the relationship between OBS and MASLD with the use of RCS curves and multivariate logistic regression. We identified a nonlinear association between OBS and MASLD (nonlinear *p* = 0.315, 0.256, 0.077) in crude models, model 1 and model 2, respectively (Figure 2). We detected that the risk of MASLD gradually decreased as OBS increased.

### Correlation between OBS and all-cause mortality

The relationship between OBS and all-cause mortality was further assessed by Cox proportional hazards models. We found that continuous OBS resulted in an 6% reduction in the risk of all-cause mortality (OR = 0.94, 95% CI 0.86–1.03; *p* = 0.204). While OBS was assessed as a categorical variable, after adjusting for covariables, the OBS Q4 group (HR: 0.15, 95% CI: 0.02–0.87; *p* = 0.035) exhibited a lower risk of all-cause mortality than the OBS Q1 group in all participants (Table 6). When adjusting for liver fibrosis, the lower risk of all-cause mortality in OBS Q4 group still exist (Supplementary Table S4), which means mortality risk might be indirectly affected by OBS itself rather than the level of fibrosis.

**Table 6:** Associations of OBS with all-cause mortality.

OBS	HR (95% CI); value of *p*					
	Crude model		Model 1		Model 2	
Continuous	0.96(0.88,1.04)	0.337	0.93(0.84,1.01)	0.096	0.94(0.86,1.03)	0.204
Q1	1.00 (ref)		1.00 (ref)		1.00 (ref)	
Q2	0.99(0.42,2.35)	0.987	0.68(0.30,1.58)	0.371	0.88(0.33,2.30)	0.789
Q3	1.82(0.50,6.67)	0.364	1.16(0.33,4.03)	0.820	1.63(0.47,5.65)	0.442
Q4	0.30(0.07,1.22)	0.092	0.17(0.05,0.63)	0.008	0.15(0.03,0.87)	0.035

Model 1: adjusted for age, gender, and race.

Model 2: Model 1+ education level, diabetes, hypertension, cancer history and serum albumin.

The OBS was converted from a continuous variable to a categorical variable (quartiles). Weighted cox regression analysis was used to obtain HR, 95% CI.

Crude model was adjusted with no covariates.

## Discussion

We assessed the association between OBS and MASLD in this large cross-sectional study on the basis of NHANES (ranging from 2017 to 2018). A 15-component-based OBS was used to extensively evaluate exposure through dietary and lifestyle-related exposure to antioxidant and pro-oxidant components, which allowed us to adequately evaluate the complexity of oxidative homeostasis in individuals. We demonstrated in this study that participants without MASLD had significantly higher OBS than those with MASLD. After adjusting for confounders, the effect of OBS on MASLD was shown to be significantly dependent on race and education level.

Extensive studies have suggested that impaired oxidative stress is associated with the occurrence and progression of MASLD/MASH through the overproduction of reactive oxygen species (ROS) to mediate lipid metabolism, insulin signaling, and innate immune signaling pathways (33–35). Either pro-oxidants or antioxidants may act antagonistically or synergistically, while antioxidants may be pro-oxidants at high doses (36). In addition, supplementation with a multitude of dietary antioxidants (vitamin E, vitamin C), as well as lifestyle changes (exercise) may improve clinical markers in part by reducing oxidative stress in NAFLD patients (37). Consequently, there is a need for a comprehensive assessment of index-OBS to evaluate oxidative balance in individuals and to investigate the impact of this balance on MASLD. Prior investigations have examined the potential benefit of this index on NAFLD through inflammation, oxidative stress, and glycolipid metabolism (38). By the way, OBS is not associated with progression of liver fibrosis. A randomized controlled clinical trial showed that vitamin E was associated with improvement in NAFLD but not liver fibrosis (39), while other studies have found that antioxidants are associated with improved fibrosis in NAFLD patients (40, 41). The reason for this consideration may be that OBS is a composite index that includes pro-oxidants and antioxidants, and therefore its combined effect cannot be explained by the alleviation of fibrosis by antioxidants alone.

The traditional OBS contains 20 items related to oxidative stress (16), but a recent study on OBS in NAFLD contained only 13 items (19). In our study, we calculated OBS contained 13 items and added caffeine intake and NSAID using to get a new OBS. In addition, there is evidence suggesting that micronutrients such as vitamins, selenium, carotenoids, and magnesium, which have antioxidant, immunomodulatory, and lipoprotective properties, may play protective roles in NAFLD (42). Polyunsaturated fatty acids (PUFA) are susceptible to lipid peroxidation implicated in NAFLD, while vitamin E has been shown to protect against non-enzymatic lipid peroxidation (43). The accumulation of excess saturated fatty acids (SFA) induces cellular lipotoxic damage and increases the risk of MASLD (44). In our study, the lower intake of antioxidant properties (carotene, vitamin C and vitamin E) and higher intake of prooxidant properties (SFA, PUFA) could be associated with the onset of MASLD. It is important that n-3 and n-6 PUFA have a differing role in oxidative stress. The n-3 PUFA have been shown to be antioxidative. However, higher intakes of n-6 PUFAS have been associated with an increased oxidative stress (45). It’s a limitation that we did not categorize PUFAs into n-3 PUFAs and n-6 PUFAs to calculate OBS.

According to our study, lifestyle may have a significant impact on the development of MASLD. In addition to dietary nutrients, there is an important role of a healthy lifestyle in avoiding MASLD. Physical activity, body mass index control and smoking cessation might be effective, which is consistent with previous studies showing the impact of obesity, sedentary lifestyle, smoking and other individual variables and situational factors on NAFLD (46). Studies showed that caffeine could improved fatty liver in ApoE KO mice with NAFLD *in vivo* (47) and NSAID-activated gene-1 (NAG-1), or growth differentiation factor-15 (GDF15), is associated with NAFLD (48). This is the first study to identify OBS included caffeine intake and NSAID as a MASLD protective factor in US adults and find a significant negative association between OBS and MASLD.

The study has both strengths and weaknesses. First, this is the first study evaluating the relationship between OBS and MASLD in US adults. Second, based on the stratified and multistage nature of the NHANES data, our findings have a high generalization among the mobile community. Third, we endeavored to account for various confounders through the use of a questionnaire and massive adjustment for variables. However, there are some limitations to this study. The present study could not effectively assess causality in the nature of the cross-sections. Although we controlled for potential confounders, we still cannot rule out the effect of unknown indexes. Another weakness of this study may be that no examination of alcohol intake was performed. However, since heavy drinkers were excluded, examination of this component was unlikely to influence the outcomes. Further validation of the predictive value of OBS for MASLD through prospective studies is needed to improve the utility of our findings.

## Conclusion

To summarize, it is suggested in this cross-sectional study that OBS is negatively associated with the incidence of MASLD and all-cause mortality in US adults. An negative association was observed between OBS and the prevalence of MASLD in a nationally representative sample of adults in the United States. Our findings confirm the role of healthy food and lifestyle choices in the prevention of MASLD. In addition, they can be used as a strategy to arrest the course of MASLD.

## Data availability statement

The original contributions presented in the study are included in the article/Supplementary material, further inquiries can be directed to the corresponding author.

## Ethics statement

The studies involving humans were approved by National Health and Nutrition Examination Survey. The studies were conducted in accordance with the local legislation and institutional requirements. The participants provided their written informed consent to participate in this study.

## Author contributions

LP: Conceptualization, Formal analysis, Funding acquisition, Validation, Visualization, Writing – original draft, Writing – review & editing. LL: Formal analysis, Investigation, Methodology, Validation, Writing – original draft. JL: Investigation, Software, Validation, Writing – original draft. YL: Conceptualization, Data curation, Funding acquisition, Project administration, Resources, Supervision, Visualization, Writing – review & editing.
